# Pandora's Box

**DOI:** 10.1192/bji.2021.28

**Published:** 2021-08

## Abstract

If you have started feeling your age or even older, stop right there! Feeling younger makes us feel better and healthier both physically and mentally; at least so say researchers from Germany. They examined longitudinal data collected over a period of three years (2014–2017) by the German Ageing Survey, with a mean age of 64 years (40–95). Controlling for baseline functional health and sociodemographic variables, they found that greater perceived stress was associated with a steeper decline in functional health, which increased with advancing chronological age. However, they also found that those who felt younger than their age showed a less steep decline in functional health and greater perceived stress was less strongly associated with functional health decline. Furthermore, they were less likely to feel stressed and this stress buffer effect was greater with increasing age.

## Acting your age is not good for you.

If you have started feeling your age or even older, stop right there! Feeling younger makes us feel better and healthier both physically and mentally; at least so say researchers from Germany. They examined longitudinal data collected over a period of three years (2014–2017) by the German Ageing Survey, with a mean age of 64 years (40–95). Controlling for baseline functional health and sociodemographic variables, they found that greater perceived stress was associated with a steeper decline in functional health, which increased with advancing chronological age. However, they also found that those who felt younger than their age showed a less steep decline in functional health and greater perceived stress was less strongly associated with functional health decline. Furthermore, they were less likely to feel stressed and this stress buffer effect was greater with increasing age.

In conclusion, feeling younger is good for your mental and physical health.

Wettstein M, Spuling SM, Cengia A, Nowossadeck S. Feeling younger as a stress buffer: Subjective age moderates the effect of perceived stress on change in functional health. Psychol Aging (2021) 36(3): 322.3393944910.1037/pag0000608

## Stop sulking

Staying with the matter of stress, here is one more tip from another study, published earlier this year. Stress is part of daily life and some of us cope better than others. Before it gets to the point where we need to book ourselves a course of CBT or mindfulness, there are simple things we can do to reduce our stress.

Researchers from Oregon State University, US, examined a sample of 2000 people, which included individuals aged between 33 and 84, who participated in a National Study of Daily Experiences. The subjects were asked to complete a daily inventory of stressful experiences, self-report measures of stress and stress resolution status, as well as daily negative and positive affect over a period of eight consecutive evenings. They examined daily negative and positive affect, avoided arguments occurring in the same day (reactivity) or the day before (residue) and whether these were affected by resolution of interpersonal stress. They found that resolution significantly dampened both negative and positive affect reactivity (the same day) and residue (the day before) associated with arguments. Negative affect was associated with avoided arguments. Older subjects were more likely to resolve both arguments they had in the same day and arguments of the previous day and had reduced reactivity associated with avoided arguments.

The lesson is that if you want peace of mind, resolve everyday arguments promptly.

Witzel DD **and** Stawski RS. Resolution status and age as moderators for interpersonal everyday stress and stressor-related affect. J Gerontol B Psychol Sci Soc Sci (2021): gbab006. doi: 10.1093/geronb/gbab006PMC859905033423065

## Beware of Narcissus

Narcissism is a term derived from the ancient Greek myth of a beautiful young man who fell in love with his own reflection, with disastrous consequences both for him and others. Narcissistic personality disorder, according to the ICD-10, is characterised by an enduring pattern of grandiose beliefs and arrogant behaviour together with an overwhelming need for admiration and a lack of empathy for, and even exploitation of, others. The personality disorder is characterized by excessive self-love, egocentrism, grandiosity, exhibitionism, excessive need for attention, and sensitivity to criticism. One can recognise many of these features in others and in those in the public eye, including some well-known politicians. These characteristics may be more sinister in some cases.

A recent study from Ohio State University, US, found a serious risk of aggression associated with narcissism. The authors carried out a meta-analysis of 437 independent studies, including a total of 123 043 subjects, with the aim of examining a link between narcissism and aggression. Indeed, they found both “normal” and “pathological” narcissism to be related to aggression in all three dimensions of narcissism (entitlement, grandiosity and vulnerable narcissism). In aggressivity they included indirect, direct, displaced, physical, verbal and bullying behaviour. The link was stronger under provocation conditions but was also present in the absence of provocation. The relationship between narcissism and aggression was significant in both genders and all ages and independent of whether they were from individualistic or collectivistic type of countries.

Kjærvik SL **and** Bushman BJ. The link between narcissism and aggression: a meta-analytic review. Psychol Bull (2021) Advance online publication. doi: 10.1037/bul000032334292012

## To fit in or not

We humans are social animals and we adhere to social norms and conventions, but how young are we when we start to conform? A recent study examined this question in very young children. The researchers invited 104 children aged 3.5 to help set up items (teas, cakes, etc.) for a tea party. The children indicated which items they preferred for the party but a quarter of them changed their mind when they listened to either an adult or another child suggest other items. These children were more likely to conform and override their own preferences when the suggestions were framed as norms (for example, “this is what we usually do at such tea parties”), rather than when they were framed as preferences. This change of mind occurred irrespective of whether the alternative option came from an adult or a child, indicating a need to follow convention rather than a response to a person in authority.

They concluded that when young children consider a certain action as conventional within their cultural group, they are motivated to conform so that they can connect to and identify with the group. The authors do not comment on the finding that three-quarters of the children did not change their mind other than saying this is understandable as they would be going against their own preferences. A little more exploration is needed on this subject.

Li L, Britvan B, Tomasello M. Young children conform more to norms than to preferences. PLoS One (2021) 16(5): e0251228.3403842010.1371/journal.pone.0251228PMC8153413

## Telling white lies

Have you ever told white lies and maybe convinced yourself you are only doing it for the good of others? Your brain may be telling a different story. A recent study using fMRI, a brain fingerprinting approach, and univariate and multivariate analyses examined the possibility that our brain could reveal selfish motivation in white lies or, as the researchers call them, Pareto lies, meaning lies that are self-serving and altruistic at the same time.

They asked participants to tell lies in order to earn a reward for themselves, for another person, or for both and they used fMRI to measure MPFC (medial prefrontal cortex) activity. Selfish motivation for Pareto lies elicited higher activity in the ventral and rostral MPFC. The ventral MPFC showed an increased 28 pattern similarity to selfish lies while the rostral MPFC showed a decreased pattern similarity to 29 altruistic lies. There were no gender differences.

These findings demonstrate that however convincing a person's behaviour is when telling white lies, the brain reveals the true motivations. Prosocial dishonesty is encoded primarily by increased activity in the specific subregions of the MPFC that are involved in valuation and strategic switching of motivation.

Kim J, Kim H. Neural representation in MPFC reveals hidden selfish motivation in white lies. J Neurosci (2021) Epub ahead of print. doi: 10.1523/JNEUROSCI.0088-21.2021.PMC826580134059555

## Don't believe everything you read!

There is a wealth of publications with an ever-increasing number of journals in social sciences but very few of these ever get replicated. Three replication projects tried to systematically replicate the findings in top psychology, economics and general science journals. The results are sobering. In psychology, only 39% of the studies yielded significant findings in the replication compared to 97% of the original experiments. In economics, 61% of 18 studies replicated and among *Nature* and *Science* publications 62% of 21 studies did. The relative effect size over the findings that did replicate was only 75% of the original findings while for the failed replications it was close to 0%. It is reassuring, though, to know that when asked to predict replication results before the replication studies, experts in the field could predict well which findings would be successfully replicated.

In a recently published study, the authors use the findings from these three replication studies in an attempt to correlate replicability with citations and to examine whether articles that failed to replicate were cited more than those that were successfully replicated, both before and after the replication projects were published.

They found that studies in top psychology, economics and general interest journals which failed to replicate were cited more often than those that were replicated. Furthermore, this difference in citation does not change after the original publication failed to replicate. Only 12% of post-replication citations of non-replicable findings acknowledge the replication failure.

Asking themselves the question, why are nonreplicable papers accepted for publication in the first place, the authors comment a possible answer is that the review team faces a trade-off. When the results are more interesting, they apply lower standards regarding their reproducibility. Pandora, in its search for interesting articles, may have fallen into the same trap!

Serra-Garcia M **and** Gneezy U. Nonreplicable publications are cited more than replicable ones. Sci Adv (2021) 7(21): eabd1705.10.1126/sciadv.abd1705PMC813958034020944

